# Diagnostic role of ultrasound elastography on lymph node metastases in patients with head and neck cancer^☆^

**DOI:** 10.1016/j.bjorl.2018.01.002

**Published:** 2018-02-16

**Authors:** Müberra Pehlivan, Melek Kezban Gurbuz, Cemal Cingi, Baki Adapinar, A. Nevbahar Değirmenci, F. Mustafa Acikalin, Mehmet Özgür Pinarbaşli, Ertugrul Colak

**Affiliations:** a Eskişehir Government Hospital, Meselik, Turkey; b Eskişehir Osmangazi University, Faculty of Medicine, Department of Otorhinolaryngology, Meselik, Turkey; c Eskişehir Osmangazi University, Faculty of Medicine, Department of Radiology, Meselik, Turkey; d Eskişehir Osmangazi University, Faculty of Medicine, Department of Pathology, Meselik, Turkey; e Eskişehir Osmangazi University, Faculty of Medicine, Department of Biostatistics, Meselik, Turkey

**Keywords:** Head, Neck, Cancer, Lymph node metastases, Ultrasound elastography, Cabeça, Pescoço, Câncer, Metástases de linfonodos, Elastografia ultrassonográfica

## Abstract

**Introduction:**

Ultrasonography is the easiest non-invasive method to diagnose lymph node metastases in patients with head and neck cancer. However, since CT scans are often preferred in the evaluation of primary tumours of these patients, information about lymphatic metastases may also available in these patients. Therefore, ultrasound is not routinely employed in the evaluation of these patients. However, elastography technique, a recent development in ultrasound technology, could make use of ultrasonography in these patients even more widespread, even though it is still not widely used today.

**Objectives:**

The aim of this study was to evaluate the role of sonographic elastography in the diagnosis of lymph node metastasis of head and neck cancer.

**Methods:**

Twenty-three patients diagnosed with head and neck cancer and scheduled for surgical treatment including neck dissection were included in the study. All patients underwent neck examination by palpation, ultrasound elastography and computerized tomography with contrast. To compare the diagnostic performance of palpation, ultrasound elastography and computerized tomography, the findings of each examination method were compared with the histopathological examination results of neck specimens.

**Results:**

15 (65.2%) patients had a primary tumour in the larynx; 7 (30.4%) in the oral cavity; and 1 (4.3%) in the parotid. 7 (30.4%) out of 23 patients underwent bilateral neck dissection. In total, 30 neck dissections were hereby taken into account during study. Ultrasound elastography showed higher accuracy (83.3%) and higher sensitivity (82.4%) than palpation and computerized tomography, but the specificity of ultrasound elastography was lower (84.6%) than palpation and computerized tomography.

**Conclusions:**

Ultrasound elastography is helpful for the diagnosis of lymph node metastases in patients with head and neck cancer. Due to its non-invasive character, it can be used safety in combination with other radiological techniques to support or improve their diagnostic performance.

## Resumo

**Introdução:**

A ultrassonografia é o método mais fácil e não invasivo para diagnosticar metástases em linfonodos em pacientes com câncer de cabeça e pescoço. No entanto, como as tomografias computadorizadas são frequentemente preferidas na avaliação de tumores primários desses pacientes, as informações sobre metástases linfáticas também se tornam disponíveis. Portanto, a ultrassonografia não faz parte da avaliação de rotina desses pacientes. Entretanto, a técnica de elastografia, um desenvolvimento mais recente na tecnologia de ultrassom, poderia tornar o uso da ultrassonografia mais difundido nesses pacientes embora atualmente ainda não seja amplamente usado.

**Objetivo:**

Avaliar o papel da elastografia ultrassonográfica no diagnóstico de metástases em linfonodos em casos de câncer de cabeça e pescoço.

**Método:**

Foram incluídos no estudo 23 pacientes diagnosticados com câncer de cabeça e pescoço e com tratamento cirúrgico programado, inclusive esvaziamento cervical. Todos os pacientes foram submetidos a exame cervical por palpação, elastografia ultrassonográfica e tomografia computadorizada com contraste. Para comparar o desempenho diagnóstico da palpação, elastografia ultrassonográfica e tomografia computadorizada, os achados de cada método de exame foram comparados com os resultados do exame histopatológico de amostras obtidas do pescoço.

**Resultados:**

Dos pacientes, 15 (65,2%) apresentaram tumor primário na laringe; sete (30,4%) na cavidade oral; e um (4,3%) na parótida. Sete (30,4%) dos 23 pacientes foram submetidos a esvaziamento cervical bilateral. Foram considerados durante o estudo 30 pescoços. A elastografia ultrassonográfica mostrou maior acurácia (83,3%) e maior sensibilidade (82,4%) do que a palpação e a tomografia computadorizada, mas a especificidade da elastografia ultrassonográfica foi menor (84,6%) do que a palpação e a tomografia computadorizada.

**Conclusões:**

A elastografia ultrassonográfica é útil para o diagnóstico de metástases de linfonodos em pacientes com câncer de cabeça e pescoço. Graças ao seu caráter não invasivo, pode ser usada com segurança em combinação com outras técnicas radiológicas para apoiar ou melhorar o desempenho diagnóstico.

## Introduction

Cervical lymph node metastasis is a major prognostic factor and the primary reason for treatment failure in patients with head and neck cancers. It is therefore important to properly evaluate the neck at the time of diagnosis in these patients. In addition to neck palpation, imaging techniques such as ultrasonography (US), computed tomography (CT), magnetic resonance imaging (MRI), and sometimes even positron emission tomography have been used for this purpose.^1^, ^2^ Among these techniques, US is the easiest non-invasive method. Technological advances that have been advancing rapidly in recent years have also positively affected the ultrasound technology. Elastography technique, a latest development in ultrasound technology, provides information about the stiffness of the tissue by measuring the degree of strain on the tissue by applying external force. Ultrasonography images are obtained with minimal pressure changes on the tissue of the ultrasonography probe. Compression leads to a shift of position, which is less in hard tissue, and it is thought that the malignant potential is increased as the tissue hardens.^3^, ^4^ Although it is still infrequently used and most of the devices in use today do not have this technology, it promises improved success in the evaluation of cervical lymph nodes in line with the results of the studies carried out so far. Even so, these studies also stressed that elastography has some unresolved issues still.^5^, ^6^, ^7^

In the present study, we also aimed to investigate whether ultrasound elastography (USE) is worthwhile to detect lymph node metastasis in patients with head and neck cancers.

## Methods

This cross-sectional study was approved by the local ethics committee with the number 14 Apr 2013/13.

### Study group

The study was comprised of patients diagnosed with head and neck cancer and scheduled for surgical treatment including neck dissection. Patients who had previously undergone neck surgery or radiotherapy to the neck for any reason were excluded from the study.

### Study design

**Figure 1:**
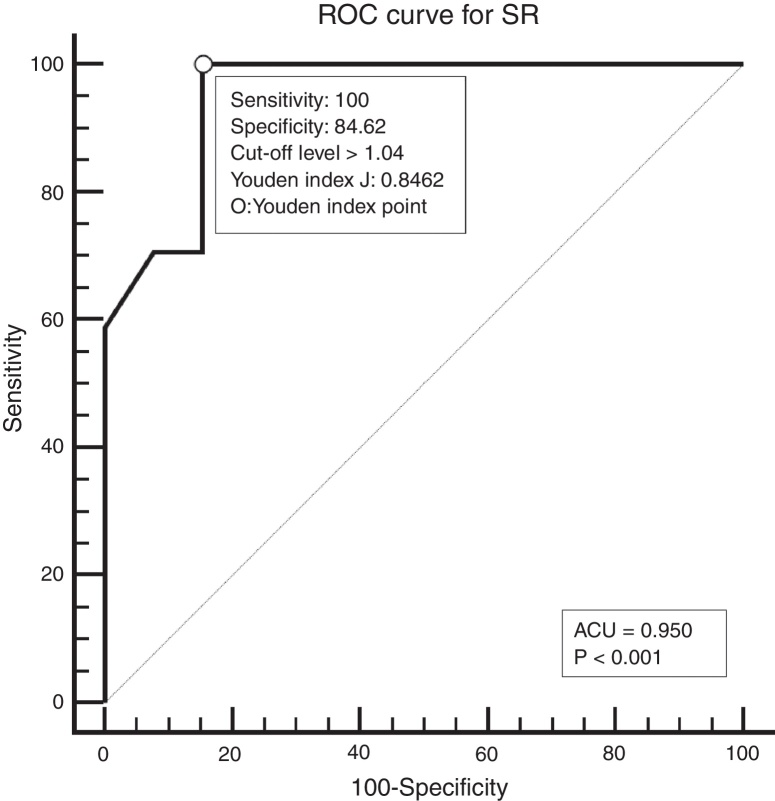
The best cut-off level founded using ROC analysis. AUC, the area under the ROC curve.

All patients underwent neck palpation, CT scan and USE preoperatively. Palpated lymph nodes larger than 1 cm, hard, immobile, attached to surrounding tissues were considered metastatic. All CT scans were performed with a 64 detector, multi-interval CT device (Aquillion 64, Toshiba, Tokyo, Japan) using contrast material, and were evaluated by an experienced radiologist. Metastatic nodes are analyzed on the basis of size, morphology, shape, margins, and distribution. Lymph nodes larger than 1 cm, having findings of necrosis, cystic change, calcification, or hyper-enhancement, having a round appearance, and located in the field appropriate for lymphatic drainage of the primary tumour were considered metastatic. All USEs were also performed by an experienced radiologist using a digital ultrasonography scanner (Hitachi EUB 7000) equipped with real time elastography software and a linear transducer of 7.5 MHz. On the static elastogram images, numerical strain values of lymph node and surrounding soft tissue were measured using ROI (region of interest). All sonograms were scored with a scoring system as shown in Table 1. The best cut-off level for SR was found to be 1.04 using ROC analysis. ROC diagram is seen in Fig. 1.

**Table 1:** USE elasticity scoring system.

Score 1	No or very small hard space
Score 2	Hard space of <45%
Score 3	Hard space of >45%
Score 4	Hard peripheral space-soft central space
Score 5	Whole is hard

All patients were staged according to results of clinical examination and radiological evaluation using TNM staging revised by American Cancer Committee in 2010. Patients having clinically *n* positive necks, and patients with clinically N0 necks with high risk of occult lymphatic metastasis (patients having early T staged tumours characterized by lymphatic spread and advanced T staged tumours) underwent neck dissection on the same side as the main tumour. For patients with bilateral lymph node metastases and having midline tumours bilateral neck dissection was performed. The type of neck dissection was determined according to the stage of the tumour. Patients with early stage tumours underwent selective neck dissection, while advanced staged patients were under gone modified radical neck dissection.

During all neck dissections, lymph nodes considered to be malignant preoperatively were marked with suture over the specimen. Individual nodes were assessed by histopathological examination.

Histopathological results were compared with physical (palpation) and radiological examination (CT and USE) findings. All statistical analyses were performed using the IBM SPSS Statistic (version 21) and MedClac (version 14.12) software packages. Diagnostic accuracy was analyzed and compared by Receiver-Operating Characteristics (ROC) curve. The area under the curve and Youden Index were used for comparison. *p*-Value of less than 0.05 was considered to indicate statistically significant difference.

## Results

A total of 23 patients (4 female, 19 men) with the average age of 56.43 ± 1.90 (ranging from 39 to 77) were included in the study. Fifteen (65.2%) patients had a primary tumour in the larynx, 7 (30.4%) in the oral cavity, and 1 (4.3%) in the parotid. The histological type of parotid tumour was mucoepidermoid carcinoma, while other all tumours were squamous cell carcinoma. Three patients (13.0%) were in early stage, and 20 (87%) patients were in advanced stage of disease according to clinic radiologic evaluation. The stage of the patient with parotid cancer was T2N1, and the detailed staging of all other patients are found in Table 2. Seven patients (30.4%) underwent bilateral neck dissection. In total, 30 necks were hereby taken into account during this study. The tumour of 4 of the patients who underwent bilateral neck dissection was in the supraglottic region of the larynx, and the stage of remaining 3 patients was N2c. In a total of 30 neck dissections, 24 (80%) were modified radical, 6 (20%) were selective neck dissection.

**Table 2:** The stages of patients except for a patient with parotid tumour.

	Larynx (*n*)	Oral cavity (*n*)
T2N0	2	1
T2N1	3	3
T3No	–	2
T3N1	3	–
T3N2c	3	–
T4N0	4	1

*n*, the number of patients.

The numbers and size of pathologic lymph nodes detected by palpation, CT-scans and USE are shown in Table 3. As seen in this table, only one lymph node was found in the neck of the patients and bilateral lymph nodes were detected in only one patient. Therefore all statistical comparisons were performed considering the number of necks with lymph nodes. Special results of USE (elasticity score [ES] and stiffness/strain rate [SR]) are also shown in Table 4.

**Table 3:** Pathologic lymph nodes detected by palpation, CT-scans and USE.

	Number of patients with lymph nodes	Number of necks with lymph nodes	Number of lymph nodes	The mean diameter of LN (min–max)
Palpation	10	12	12	1.5 (0.5–4) cm
CT-scans	11	13	13	1.2 (0.3–4) cm
USE	13	16	16	1.3 (0.2–4) cm

**Table 4:** The results of USE.

		Number of lymph nodes
ES	1	–
	2	4
	3	10
	4	–
	5	2
SR	≤1.04	11
	>1.04	5

ES, elasticity score; SR, stiffness/strain rate.

Seventeen (in 13 patients/17 necks) metastatic lymph nodes with the mean diameter 1.6 (min 0.4 – max 4) cm were detected by histopathologic examination.

The comparison of physical examination and radiologic imaging results with histopathologic results is shown in Table 5.

**Table 5:** The comparison of physical examination and radiologic imaging results with histopathologic results.

	A	B		Sensitivity	Specificity	Accuracy (A)	PPV	NPV
		No	Yes					
Palpation	No	9	9	47	92	66.7	0.88	0.57
	Yes	4	8					
CT scans	No	12	5	70.6	92.3	80.0	0.92	0.70
	Yes	1	12					
USE	No	11	3	82.4	84.6	83.3	0.87	0.78
	Yes	2	14					

A, the number of necks with/without pathological lymph nodes; B, the number of necks with/without histopathological positive lymph nodes.

## Discussion

The basic general approach to the treatment of patients with head and neck cancer is as follows: if the patient is at an early stage, treatment using a single treatment modality (i.e. surgery or radiotherapy) is chosen, and a patient with advanced stage is treated by a combined treatment modality including surgery and radiotherapy. However, one of the most important factors determining the type and the extent of treatment is occult or detected nodal metastases. So, it is recommended by some authors that patients having a high risk of occult lymph node metastases or clinically *n* positive patients be treated surgically, including neck dissection to clarify the stage of tumour by histopathologic examination. Otherwise, if radiotherapy is a treatment option, it should include lymph node areas in the neck associated with tumour spread. For this reason, detailed neck evaluation is essential in these patients, and the basic method suggested for this purpose is attentive neck palpation involving all cervical anatomic triangles. However, a clinical palpable lesion must be 0.5 cm for superficial regions and minimum 1 cm for deeper regions. True detectability rate (accuracy rate) of neck metastasis by palpation has been reported around 70%.^8^, ^9^, ^10^, ^11^ In our study, the sensitivity, specificity and accuracy rates of palpation were 47%, 92% and 66.7% respectively. These values are certainly quite insufficient to make accurate assessments. Therefore, advanced radiological imaging methods are needed for detailed evaluation of neck. Today, the most preferred imaging methods include CT, MRI, US and PET-CT for this purpose.^1^, ^2^, ^8^, ^9^, ^12^ Among these, the most used modalities are CT and MRI, because the primary tumour is already evaluated with them. United Kingdom National Multidisciplinary guidelines also states that CT or MRI is mandatory for staging neck disease in head and neck cancer with choice of modality dependent on imaging modality used for the primary site, local availability and expertise.^13^ However, these techniques can also lead to false negative or positive prediction. In a meta-analysis published in 2012, on detection of cervical lymph node metastases in head and neck cancer, patients with clinically N0 neck, sensitivity-specificity of CT and MRI were reported as 52–93% and 65–81%, respectively.^14^ Therefore, in cases whose lymph node metastases cannot be clearly elucidated, an additional technique for proper tumour staging is necessary to make optimal treatment plan. The classical approach in such cases is to perform PET-CT with a sensitivity of 77–96% and a specificity of 82–100%.^15^, ^16^ In fact, this technique is more sensitive for detecting synchronous second primaries and distant metastases, and is an expensive, invasive method using radioactive substances that emit positron rays. For this reason, ultrasound can be preferred as an alternative, thanks to available, cost effective and noninvasive properties to detect cervical lymph node metastases in head and neck cancers. Also, conventional US imaging was recently enhanced using elastography techniques, based on the consideration of whether the tissue is hard or soft, allowing diagnostic information about the presence or status of disease. Namely, the elastography technique gives information about the elasticity and flexibility of tissues, and it is established based on the hypothesis that soft tissues deform more than hard tissues. So, in accordance with idea that cancerous tissue will often be harder than the surrounding tissue, distinction between benign and malignant lesions can be made more accurately.^5^, ^7^, ^17^ It has also been shown in the literature elastography improves the diagnostic performance of conventional ultrasonography by increasing the Negative Predictive Value (NPV) of conventional ultrasonography.^7^, ^17^, ^18^

Today, two basic elastography techniques are available: hand free technique and shear wave technique. In both techniques, a lesion previously identified with B-mode US is characterized by its elasticity. The free technique is cheaper, faster to perform, easier to learn, and more widely used, but the practical disadvantage is that it is user-dependent. The fact that the pressure applied by the probe is not standard causes wide variability between magic and elasticity values in this technique. To prevent this, scales showing the amount of compression applied and stimulating the user are created in devices. Additionally, semi quantitative strain rate measurement “strain ratio” exhibiting elasticity and reducing variability, has been improved in hand free elastography. This method is called as “strain elastography”. Despite these precautions, unfortunately, variability is always a major problem for this technique. However, in shear wave elastography, instead of external compression applied by ultrasound probe, high powerful (frequency 2.67 MHz) acoustic impulse radiation force causes little substitutions (1–10 μm) in the tissue when applied. In this way, mild compression of probe is sufficient and the user-dependent variability has been eliminated in this technique.^17^, ^19^, ^20^, ^21^

In this study, real time strain elastography was performed. There is only one reason for this preference: it is the characteristic of the ultrasonic device that exists in our hospital. Nevertheless, it was a great chance for us to work with an experienced radiologist in applying this technique, as evidenced by high accuracy rate of 83.3% and high sensitivity rate of 82.4% we achieved. Our inaccuracy rate of 16.6% may be because as stated in the literature lymph nodes that are not wholly infiltrated by cancer cause confusion, since elasticity scores or strain ratios evaluate stiffness with reference to the entire node.^20^ Additionally, our result that USE has been found to have a slightly (around 3%) higher accuracy rate than CT encouraged us using USE, especially when we cannot clearly elucidate the lymph node metastasis in patients whose treatment decision will be clear according to the neck disease. Nevertheless, the small number of patients in our study limits our impressions, and further more extensive studies are required to confirm our results. For this reason, physicians should encourage the training and use of elastography.

## Conclusion

Ultrasound elastography is helpful for the diagnosis of lymph node metastases in patients with head and neck cancer. It can be used safety in combination with other radiological techniques to support or improve their diagnostic performance thanks to its non-invasive character.

## Conflicts of interest

The authors declare no conflicts of interest.
